# Genome Sequence of *Pseudomonas* sp. Strain ST1, Isolated from Olive (Olea europaea L.) Knot Galls in Croatia

**DOI:** 10.1128/MRA.00986-19

**Published:** 2019-11-14

**Authors:** Gabriela Vuletin Selak, Marina Raboteg, Pascale Fournier, Audrey Dubost, Danis Abrouk, Katja Žanić, Slavko Perica, Philippe Normand, Petar Pujić

**Affiliations:** a Institute for Adriatic Crops and Karst Reclamation, Split, Croatia; b Ecologie Microbienne, Centre National de la Recherche Scientifique UMR 5557, Université de Lyon, Université Claude Bernard Lyon I, INRA, UMRA1418, Villeurbanne, France; University of Arizona

## Abstract

We report the genome sequence of a *Pseudomonas* sp. strain isolated from olive knot galls. The genome size is 6.101 Mbp with a G+C content of 58%. A total of 6,137 coding DNA sequences (CDS) were predicted, including 52 tRNA and 4 rRNA genes.

## ANNOUNCEMENT

Olive knot is one of the most important diseases of the olive crop and is present in all olive-growing regions. Pseudomonas savastanoi pv. savastanoi is the causal agent of olive knot disease, which results in tumorous overgrowths (knots). This olive knot pathogen bacterium can survive and multiply on aerial plant surfaces as well as in knots. The earlier studies of Pseudomonas savastanoi pv. savastanoi virulence implicated the type III secretion system, phytohormones, and quorum sensing (QS) as being involved in the disease process (1, 2). The pathogen can be dispersed both within the plant and to surrounding plants in wind-blown rain, by insects, and by human activities, entering the plant through wounds. Populations of *P. savastanoi* are normally associated with nonpathogenic bacteria, both epiphytically and endophytically (1, 3, 4). More specifically, the disease progression and knot volume were increased by coinoculation of Pseudomonas savastanoi pv. savastanoi with nonpathogenic bacteria (1, 4, 5).

*Pseudomonas* cells were isolated from olive knots from olive plants grown in the central region of Dalmatia, Croatia (43°30′19.6ʺN, 16°29′55.0ʺE). Olive knots were harvested from a plant trunk using a sterile scalpel, immediately surface sterilized using 75% ethanol, and sliced using a sterile scalpel, and the slices were placed on the surface of King’s medium agar plates (6). Plates were kept in the dark at 25°C for 48 hours. Cells from a single fluorescent colony identified under UV light on an agar plate were transferred into 10 ml of liquid lysogeny broth (LB) medium in a 50-ml Falcon tube and grown at 28°C for 24 hours with shaking. Genomic DNA was isolated from bacterial cells using a microbial DNA kit (reference number 740235; Macherey-Nagel, Hoerdt, France). The 16S RNA gene was amplified with PCR using 20 ng genomic DNA, 0.2 mM deoxynucleoside triphosphates (dNTPs), 50 nM each com1 (5′CAGCAGCCGCGGTAATAC) and com2 (5′CCGTCAATTCCTTTGAGTTT) primers, and 2.5 U *Taq* polymerase (Invitrogen, France). The amplified DNA product was sequenced using Sanger sequencing at Biofidal, Vaulx-en-Velin, France. Sequence comparison using a BLASTN search with the NCBI database showed that the strain belongs to a group of the genus *Pseudomonas*. The *Pseudomonas* sp. strain ST1 genome bank was made using a Nextera XT DNA library prep kit and protocol (Illumina, Évry, France) and sequenced using Illumina MiSeq technology with a paired-end 2 × 300-bp run (Biofidal). Quality controls were made with FastQC (7) and Trimmomatic (8). We obtained a total of 8,334,104 reads with 416× coverage, an *N*_50_ value of 0.082 Mbp, and a G+C content of 58%. Default parameters were used for Unicycler assembly with a minimum contig length of 200 bp. Genome assembly was performed using Unicycler version 0.4.3, and annotation was done with the MicroScope platform version 3.10.0 (9, 10) using the Rapid Annotations using Subsystems Technology (RAST) (11) and PATRIC (12) Web servers and Prokka software (13). The genome of *Pseudomonas* sp. strain ST1 has 6,070,031 bp assembled in 318 contigs. The genome has 6,019 predicted genes. The public version of the *Pseudomonas* sp. strain ST1 genome sequence at GenBank was annotated using the Prokaryotic Genome Annotation Pipeline (PGAP) (14). The version described in this paper is the first version.

### Data availability.

The complete genome sequence described here has been deposited in NCBI/GenBank under BioProject number PRJNA555035, BioSample number SAMN12289196, accession number VKOF00000000, and SRA number SRX6799169.
